# Prevalence of Proteinuria in Owned Dogs from Italy: A Multicentric Study

**DOI:** 10.1155/2019/6073624

**Published:** 2019-03-19

**Authors:** M. Gizzarelli, X. Roura, P. Scarpa, P. D'Ippolito, V. Foglia Manzillo, G. Oliva, A. Tarducci, A. Borrelli, G. Melis, F. Quintavalla, A. Uva, A. Guarraci, A. Zatelli

**Affiliations:** ^1^Department of Veterinary Medicine and Animal Production, University of Naples Federico II, Naples, Italy; ^2^Hospital Clínic Veterinari, Universitat Autònoma de Barcelona, Bellaterra, Spain; ^3^Department of Veterinary Medicine, University of Milan, Milan, Italy; ^4^R&D Department, Medical Consultancy Services, Ta'Xbiex, Malta; ^5^Department of Veterinary Sciences, University of Turin, Grugliasco, Italy; ^6^Department of Veterinary Science, University of Parma, Parma, Italy; ^7^Ospedale Veterinario Pingry, Bari, Italy; ^8^Clinica Veterinaria San Lorenzo, Palermo, Italy

## Abstract

Even though proteinuria is related to different causes, when it is persistent and associated with inactive urinary sediment, it is primarily due to kidney disease. Early detection of proteinuria allows us to identify several pathological conditions. The aim of the study was screening a canine population not known as being proteinuric, by the urinary dipstick. The study was carried out in seven Italian veterinary clinics during a period of six weeks. Dogs were enrolled with no restriction of sex or age. Females in estrus, dogs with signs of genitourinary diseases, or those previously diagnosed with proteinuric nephropathy were excluded. Dogs were considered “nonproteinuric” (NP) in case of negative dipstick test or “suspected proteinuric” (SP), if positive at the dipstick. When possible, proteinuria was confirmed by UPC ratio. A total of 1156 dogs were evaluated: 414 were from northern Italy and 742 from southern Italy. Based on dipstick test, 655 (56.6%) dogs were NP, while 501 (43.3%) were SP. Among the NP dogs 225 out of 414 (54.3%) were in northern Italy and 430 of 742 (57.9%) in southern Italy. One hundred eighty-nine of 414 (45.7%) SP dogs were identified in northern Italy and 312 of 742 (42.1%) in southern Italy. No statistical difference was found between the North and the South of Italy. UPC was available in 412 out of 501 SP samples: proteinuria was confirmed in 263 (63.86%) samples. Results from our study showed a high percentage of suspected proteinuric dogs, apparently not affected by renal diseases, together with the absence of statistically significant differences based on geographical area.

## 1. Introduction

Proteinuria is defined as the presence of an excessive amount of proteins in the urine, or of abnormal proteins that are not normally present [1–4]. Over the last years, canine proteinuria has assumed a position of prominence and growing interest enough to be now considered not only a marker of nephropathy, but also a prognostic factor [2–5]. Proteinuric dogs, for the same IRIS stage, show a different progression of the nephropathy depending on the amount of protein lost in the urine and they have an increased risk of mortality due to renal causes compared to nonproteinuric dogs [2–7]. Moreover, the response to the antiproteinuric therapy influences the progression of the disease: the possibility of instituting an antiproteinuric therapy, associated with the early diagnosis of proteinuric nephropathy, has proved to be effective in reducing the risk of mortality due to renal causes in several natural models of the disease [3, 4, 7].

Causes of proteinuria of renal origin include either genetic or infectious diseases, other than all diseases leading to a chronic stimulation of the immune system and immune-mediated disorders in general. Many of the underlying conditions of the proteinuric nephropathy are not identified or are identified belatedly and this makes the management of the renal disease difficult; furthermore, the lack of easily identifiable clinical signs often makes the diagnosis late.

Being the presence of proteinuria a marker of nephropathy as well as an important prognostic factor in dogs affected by kidney disease, screening of the population at risk can assume a role of particular importance as most proteinuric dogs are not identified at the early stages of the disease for the lack of clinical signs [3, 4, 7, 8]. In order to carry it out successfully, the screening of the canine population not only requires the identification of dogs at risk of the disease, but also it is necessary that the tests chosen are easy to use, of low costs, and performed on samples collected with no invasive procedures. In dogs, the urinary dipstick for the identification of nonproteinuric patients has been proposed [2, 9, 10]. Dogs whose urine is negative at the dipstick can be considered nonproteinuric, while all samples with positive results should undergo the more sensitive and specific urinary protein to creatinine ratio (UPC) [10]. Urine collection by spontaneous micturition (free catch) has also been validated in dogs [8, 11]. Positive results at dipstick will require us to rule out any underlying inflammatory process (through the urinary sediment evaluation), which will finally allow us to identify false positive results [8, 11]. Prerenal causes of proteinuria determining positive results at the dipstick are more commonly related to easily identifiable clinical pictures, such as fever and seizures.

The causal relation between some diseases determining a chronic stimulation of the immune system and glomerular disease causing proteinuria in dogs is suspected. Chronic disease of the skin, as well as periodontal disease, may in fact represent aetiologically important factors which could considerably widen the number of dogs at risk of proteinuria [12, 13].

Since the presence of proteinuria can be associated with genetic disease or else determined by several diseases causing glomerular damage on immune-mediated basis and the proteinuric renal disease is difficult to be identified (especially in its early stages) if not through laboratory tests, the identification and the screening of the population at risk play a key role for the early diagnosis of proteinuric nephropathy [3, 4, 6, 14]. Breeds at risk of renal disease for genetic reasons should be screened early (within 6 months of age), and all dogs of medium-senior age would deserve a renal function evaluation together with a urinalysis at least every 12 months [15].

The canine population at high risk of proteinuric nephropathy, for example, because of infectious diseases, should be screened every 6 months.

Given the multiple conditions able to determine proteinuria in dogs, epidemiological data represent a key factor in setting up a screening of the population; based on high prevalence of the disease, screening could be addressed not only to breeds at risk for developing proteinuria or categories potentially at risk such as dogs affected by vector-borne diseases (e.g., leishmaniosis, ehrlichiosis, anaplasmosis, or borreliosis), but also to the entire canine population. Moreover, epidemiological studies carried out in Belgium on a healthy canine population revealed a high prevalence of renal disease, thus leading the authors to propose the screening of all senior dogs, even those considered healthy, to allow the diagnosis of renal disease otherwise not identifiable at the clinical visit [16]. In Italy, data regarding the prevalence of proteinuria in dogs are not available.

Main aim of the present study was screening a canine population, living in Italy and not known as being proteinuric, by the urinary dipstick. When available, the UPC values obtained from the urine of dogs positive at the dipstick were evaluated to confirm or exclude proteinuria and to determine its prevalence in the screened population.

## 2. Material and Methods

The study was conducted in Italy over a period of 6 weeks. Seven clinics participated and enrolled the dogs: 4 were located in northern Italy (Milan, Turin, Parma, and Genoa) and 3 in the south of Italy (Naples, Bari, and Palermo). All dogs not known as being proteinuric, regardless of the age or gender, were included. Females in oestrus and dogs known or suspected to be affected by diseases of the genitourinary tract and/or from any diseases determining proteinuria were excluded. Dogs in treatment with drugs influencing the level of proteinuria were also excluded from the study. All owners were informed of the aims of the study and no dog underwent invasive urine sampling (cystocentesis), if not required by the practitioner for a diagnostic work-up not related to the study.

### 2.1. Urine Collection and Storage of the Urine Samples

All urine samples were collected by free catch or cystocentesis [8, 11]. The urine samples were then stored in a dedicated container, kept at room temperature, and examined with the dipstick (Mindray®) within one hour from collection.

When possible, urinary sediment of samples positive to the dipstick was evaluated and, if inactive, the UPC was then determined. All urine samples undergoing the UPC determination were immediately centrifuged at 1500 rpm for 10 minutes and the supernatant was then separated and put into sterile tubes at −20°C, if the UPC was not performed within 6 hours from collection [17]. To measure the UPC ratio, each clinic applied a standardized technique among the ones already validated for the purpose.

### 2.2. Classification of the Results of the Urinary Test

Based on the dipstick results, dogs were considered either [10]

- nonproteinuric (NP): dogs with negative result at dipstick, or
- suspected proteinuric (SP): dogs in which dipstick gave any positive results (any colorimetric changes).

When the UPC determination was available, dogs classified as SP were further divided into three different substages in accordance with the IRIS staging system [6]:

- Nonproteinuric (NP): dogs with UPC less than 0.2
- Borderline proteinuric (BP): dogs with UPC between 0.2 and 0.5
- Proteinuric (P): dogs with UPC above 0.5.

### 2.3. Statistical Analysis

The statistical analysis was performed using MedCalc software (Frank Shoonjans, V.7.2.1.0) and SPSS, version 13.0 for Windows. Differences in prevalence between different sites were assessed by the two-sided chi-square test t. A P value <0.05 was considered statistically significant.

## 3. Results

During the period of the study, a total of 1156 dogs were screened for proteinuria, with no homogenous distribution among the clinics: 414 dogs were screened in northern Italy and 742 in southern Italy. Based on dipstick results 655 (56.6%) dogs were NP, while the remaining 501 (43.3%) were SP. Among the NP dogs 225 out of 414 (54.3%) were in northern Italy and 430 of 742 (57.9%) in southern Italy. One hundred eighty-nine of 414 (45.7%) SP dogs were identified in northern Italy and 312 of 742 (42.1%) in southern Italy. The results of the screening with the urinary dipstick are reported in Table 1, where the screened population was subdivided based on the geographical distribution and the test results as NP or SP.

A comparison was made between the two distributions relating to the percentage of negativity and positivity to the screening test performed by the dipstick and no statistical difference was found between the North and South of Italy.

UPC was available in 412 out of 501 SP samples at dipstick. Based on the IRIS staging system (IRIS, 2015), 128/412 (31.0%) were in substage BP, 135/412 (32.8%) were in substage P, and 149/412 (36.2%) were in substage NP. In total, UPC confirmed proteinuria of any degree in 263 (63.86%) of 412 urine samples analysed.

Although complete data were available only for 391 dogs from 2 veterinary clinics located in northern Italy and one in the south, there were no statistically significant differences between dogs younger or older than 6 years of age (p>0.05).

## 4. Discussion

Based on dipstick results, the present study was suggestive of low prevalence of nonproteinuric dogs and high prevalence of suspected proteinuric ones from an apparently not-renal affected population (not known to be nephropathic). These results agree with a previous article that revealed a high prevalence of proteinuria in dogs of advanced age and geriatrics [16]. An interesting aspect of this study resided in the characteristics of the canine population subjected to screening, because all dogs were considered to be apparently clinically healthy. The authors, confident that often the clinical signs of renal disease are subtle and difficult to identify, concluded by recommending measurement of proteinuria as part of geriatric health screening [16]. The same recommendation had already been provided by the American Animal Hospital Association (AAHA) for dogs of mature age and seniors [18]. The AAHA suggestions lead us to lower the age in which to begin the screening of the canine population for kidney disease compared to what was suggested by Marynissen and colleagues [8]. AAHA guidelines, in fact, consider these dogs “mature,” which have reached 50% of the expected lifespan. Similarly, the International Renal Interest Society (IRIS) recommends the screening for renal disease once a year for dogs of advanced age, even if they are apparently healthy [6]. The IRIS also recommends a renal screening every 6 months for all dogs at risk of renal disease including in this category dogs affected by diseases known to determine renal damage [6]. Despite the fact that most of the vector-borne disease are to be encountered as causing renal damage, a role of primary importance (at least for the prevalence in the general canine population) could be attributed to conditions potentially capable of determining a chronic stimulation of the immune system, such as periodontal disease and certain skin diseases [12, 13].

Suggestions provided by AAHA, IRIS, and other authors must be taken in either the social realities or the geographical locations where dogs live, as indications of screening from a certain age and over may be not applicable in all contexts, because of the presence of diseases potentially predisposing the onset of renal damage [3, 4, 6, 14, 16, 18]. The suggestion of screening the entire canine population to promote an early diagnosis of proteinuric renal disease implies the assessment of three elements: (i) the possibility to modify the prognosis thanks to an early diagnosis and an effective treatment, (ii) the possibility to perform the screening by a noninvasive sampling and a low cost exam, and (iii) high prevalence of the disease.

Proteinuric nephropathy is a disease usually at slow progression (thus, a long-term one) and high incidence (particularly, in certain geographical areas endemic, for example, for vector-borne diseases) and for the high prevalence of other concomitant illnesses, such as, for example, chronic cutaneous and periodontal disease. When epidemiologically studied, both slow progression and high incidence are the two main characteristics of diseases at high prevalence. In Spain, a prevalence of proteinuric renal disease ranging from 30.2% to 52.7% has been reported in the canine leishmaniosis population [19]. The high prevalence of proteinuria reported by the Spanish authors can obviously be related to leishmaniosis, which is well known to cause glomerular damage and consequently proteinuria. Italy, as a country with different geographical areas endemic for vector-borne diseases (e.g., ehrlichiosis, anaplasmosis, and leishmaniosis), could be epidemiologically comparable to Spain. Results of our screening allowed us to classify only 56.6% of the population of dogs as NP, which is a very low prevalence considering the fact that the population tested was supposed to be healthy. Furthermore, our data confirmed extremely high prevalence of proteinuria with 43.3% of the tested dogs as SP at the urinary dipstick, with 24.6% confirmed to be P or BP at the UPC determination. However, this study has also revealed no difference in the prevalence of nonproteinuric dogs in northern Italy compared to the south of Italy. This is not in line with what we had expected, the areas of southern Italy being characterized by a higher prevalence of vector-borne diseases (potentially determining renal damage) than the areas of northern Italy [20]. Many could be the reasons for the data obtained, which show no statistical difference between the prevalence of proteinuria in endemic and nonendemic areas for vector-borne diseases. First of all, it should be mentioned that many dog owners who live in the north of Italy used to move to the coastal areas and the south of Italy over summer, where endemic diseases transmitted by vectors are widespread. Despite the use of vector repellents and ectoparasiticides, there is the likelihood that dogs staying for even short periods of time in endemic areas contract infections that hesitate in seroconversion and subsequent production of circulating immunocomplexes, with final renal damage. The relevance of the movement of dogs via the rescue associations, which have become particularly numerous in recent years and frequently characterized by the movement of dogs from southern Italy to the north, should also be stressed. Coexisting diseases potentially causing renal damage could represent a further element. Undoubtedly, the risk of periodontal disease in dogs does not recognize geographical differences (or, at least, we are not aware of this so far), and other diseases may have contributed to determine a uniform prevalence in the proteinuria distribution on Italian territory. A final interesting data is the lack of statistically significant difference based on age (p>0.05). Even if it is limited to only three clinics of the seven enrolling the dogs to be screened, there was in the canine population ages less than or equal to/over 6 years.

This study has several limitations. First of all, the screening of the canine population has been carried out using the urinary dipstick; a test with greater sensitivity and specificity could have provided more accurate results; however it is undoubted that a negative result at the dipstick is reliable enough to identify dogs to be considered with high probability of NP [9, 10]. Another important limitation is represented by the multicentric structure of the trial, which led to the use of different methods for the determination of the UPC, as all clinics involved in the study used their own accredited method. A recent study by Rossi G. and colleagues showed how analytic variability and method-dependent differences could affect substaging of patients according to the International Renal Interest Society (IRIS), particularly regarding the values of UPC close to the cut-offs [21]. It is also worth mentioning that the same author also demonstrated the same method but different reagents can further influence a correct substaging of the patient. [22]. These two potential biases resulting from the determination of the UPC should in any case be considered as belonging to the clinical practice. The lack of statistically significant difference in the prevalence of proteinuria among the structures participating in the study could be consequent to the repetition of bias over the involved structures. Finally, a further limitation is represented by the selection of the population. For enrolment, the canine population had to be considered as apparently not affected by diseases capable of determining proteinuria. Thus, the capacity of the veterinary surgeon had a fundamental role in the selection of cases, as no laboratory examination could be used to further classify the dogs. Although there may be some bias, it is also true that these are the same encountered in daily practice. Moreover, considering the prevalence of proteinuria based on age, a limitation can be represented by the arbitrary subdivision based on age, which does not consider size and/or breed of dog. In fact, a dog belonging to a breed with an average lifespan of 9 years has passed 50% of the expected lifetime at 6 years, while a dog with an expected lifespan of 15 years has got only 40% of the expected lifetime at 6 years [18]. Of course, the risk of kidney damage also increases in relation to age and since no data about either breed or size of the population studied is available, it was not possible to evaluate a possible correlation between positivity/negativity with the urinary dipstick and the biological age of the dogs. It is possible that in some clinics dogs of advanced biological age have prevailed on those of younger biological age. The fact that among the three clinics in which complete data about the age of the dogs subjected to screening were available significant differences did not come out may suggest that dogs of different breeds and/or sizes as well as different ages (biological also) may have been well represented. Last but not least we do not evaluate the serological positivity for vector-borne disease of dogs, which underwent the screening for proteinuria, and this makes it impossible to assume a correlation between infectious disease and proteinuria itself in the population studied and the possible enrollment of subclinical patients.

## 5. Conclusions

The results of this study are suggestive of a low prevalence of nonproteinuric dogs with a high prevalence of suspected proteinuric dogs, together with the absence of statistically significant differences based on geographical area, for proteinuria of dogs living in Italy. These results also suggest proposing periodical screening, possibly annually. All dogs undergoing the screening were considered as not affected by diseases capable of determining proteinuria by skilled veterinary surgeons, based on the sole clinical examination; results of this study testify problems related to the correct identification of the proteinuric disease solely on the basis of the clinical visit and history of the dog.

## Figures and Tables

**Table 1:** Dogs classified as non- or suspected proteinuric on the basis of the screening carried out with reactive strip for urine. In this table the absolute numbers for geographical distribution and the relative percentages to facilitate the comparison of results for the different geographical areas are reported.

	Number of dogs	Nonproteinuric dogs	Suspected proteinuric dogs
Northern Italy	414	225/414 (54.3%)	189/414 (45.7%)
Southern Italy	742	430/742 (57.9%)	312/742 (42.1%)
Italy	1156	655/1156 (56.7%)	501/1156 (43.3%)

## Data Availability

The raw data used to support the findings of this study are available from the corresponding author upon request.
